# Breeding density affects the movements of gull chicks, the size of their home ranges and their association with neighbours

**DOI:** 10.1098/rsos.231431

**Published:** 2024-05-01

**Authors:** R. Salas, W. Müller, E. Stienen, H. Matheve, B. Vanden Broecke, F. Verbruggen, L. Lens

**Affiliations:** ^1^ Behavioural Ecology and Ecophysiology Research Group, University of Antwerp, Antwerp 2610, Belgium; ^2^ InnovOcean Campus, Flanders Marine Institute, Ostend 8400, Belgium; ^3^ Terrestrial Ecology Unit, Ghent University, Ghent 9000, Belgium; ^4^ Research Institute for Nature and Forest, Brussels 1000, Belgium; ^5^ Department of Experimental Psychology, Ghent University, Ghent 9000, Belgium

**Keywords:** social network, developmental plasticity, *larus*, colonial breeding, movement ecology

## Abstract

Colonies of ground-nesting species often have heterogeneous nest densities and their offspring experience different social conditions depending on the size and location of the breeding territory. For example, unintentional territory crossing by mobile chicks can trigger strong aggression from neighbouring adults, as observed in semi-precocial gulls. This would be expected to shape chicks’ movement tendencies, exploratory behaviour and propensity for social contact through aversive feedback learning or pre-natal maternal effects, as mothers may pre-adapt their offspring’s behaviour to the expected early life conditions. Therefore, we hypothesize that lesser black-backed gull chicks reared in denser areas of the breeding colony will move less, have smaller home ranges and have fewer social contacts with chicks from neighbouring nests. To test this, we first cross-fostered full clutches between and within high- and low-density parts of the colony, and then used ultra-wideband tags to track free-ranging chicks. In line with our predictions, we found that chicks reared in denser areas had a lower movement activity and smaller home ranges. However, these chicks still had more social contacts, although not necessarily with a higher number of unique individuals. Pre-natal breeding density had no significant effect on any of the parameters. We conclude that parental nest choice strongly affects the early social environment of their chicks, which can shape the development of their (social) phenotype, with potentially long-lasting consequences.

## 1. Introduction

Colonial breeding is a widespread phenomenon in nature [1–3]. Colonial aggregation during the breeding season offers several advantages, such as easier access to potential mates [4–6], greater foraging efficiency as colonies can act as ‘information centres’ [7–10] or better defence against predators [11–16]. However, colonial breeding also incurs costs associated with competition for nest sites [17,18], depletion of food resources in the vicinity of the colony [19–22] and, at least in some species, predation by conspecifics on eggs and nestlings [23].

Colonial breeding is particularly prominent in seabirds, where approximately 95% of species breed in colonies [24]. In most species, the above costs and benefits of colonial breeding have usually been studied from the perspective of the adults, although their offspring may bear considerable costs of growing up in a colony. This hypothesis is still poorly supported by data. In ground-nesting colonial species, where direct contact between offspring from neighbouring nests can be common, the social component of the early life environment may be particularly relevant. Consequently, the behaviour or phenotype of an individual may be directly influenced by the actions of other conspecifics, implying that individuals are both agents and targets of selection (interacting phenotypes *sensu* [25].

Yet, colonies are highly heterogeneous environments, with central areas of high nesting density typically surrounded by more dispersed, isolated territories [26]. This variation in nest density within colonies can lead to large variation in the social conditions to which offspring are exposed. Thus, by establishing a breeding territory within a colony, parents indirectly select the future social environment of their young, which can have important consequences for their behavioural development during the post-natal period. For example, for the highly mobile, semi-precocial chicks of *Larus* gulls, accidentally crossing the territorial boundary can provoke strong aggression from neighbouring adults [27,28], and such interactions, sometimes fatal for the chick, are expected to occur more frequently when densities are higher [17,29–31].

To cope with a hostile social environment early in life, chicks may modify their behaviour. On the one hand, lower movement activity would reduce the likelihood of crossing territorial boundaries, and as such, reduce the probability of an aggressive encounter. Consistent with this idea, we have recently shown that gull chicks reared at high breeding densities in a colony exhibit less exploratory activity in novel environments [32]. Furthermore, the early-life social environment may affect resilience to (social) stress [33] or anxiety, with the latter again facilitating avoidance of aggressive interactions [33,34] (but see [32]). On the other hand, the early experience of aggression may enhance the expression of aggressive behaviour [35], which in turn may increase a chick’s competitive ability, for example, by upregulating testosterone production (i.e. similar to the mediation of aggression and facilitation of larger territory acquisition observed in adult yellow-legged gulls (*Larus cachinnans*) [36]). Despite the differences between the various accounts, they all predict that chicks will adapt their behaviour to the social environment during early life.

Although parents largely determine the future post-natal social environment of their offspring when they establish a breeding territory, chicks do not necessarily hatch unprepared. Indeed, mothers can allocate components, such as androgens, into their eggs based on the social environment they are experiencing during egg laying [37–39], which are thought to prepare their chicks for the expected post-natal conditions [40–42]. For example, maternal yolk androgens have previously been associated with activity levels [43], exploratory behaviour [44] and territorial behaviour [41], and as such, can be hypothesized to help prepare chicks for the expected post-natal (social) conditions [40–42].

In this study, we examine the effects of the early-life social environment on movement activity, home range size and social associations with neighbours in chicks of the lesser black-backed gull (*Larus fuscus*), a semi-precocial species whose chicks begin to move at approximately 5–7 days of age. Early life movement forms the basis of exploratory behaviour, which has also been shown to be influenced by the early social environment [32], and thus potentially the level of aggression to which chicks are exposed. While until recently it was virtually impossible to study the movements and social interactions of chicks under natural conditions, recent developments in tracking technology now allow one to follow free-ranging individuals with high temporal and spatial accuracy. This allows us to test whether and how movement behaviour, home range size and social associations during early life are influenced by the spatial proximity to other neighbouring territories. To this end, chicks were measured and subsequently tracked with ultra-wideband (UWB) tags for three consecutive days towards the end of the nestling period. We cross-fostered entire clutches between (and within) areas with different breeding densities prior to tracking, to separate direct phenotypic effects of the post-natal environment from indirect, pre-natal maternal effects that may predetermine chick movement, home range size and social associations. We hypothesize that chicks hatched from clutches laid in high-density areas, where neighbouring nests are more densely packed and social encounters are more likely, or reared there, will have smaller home ranges and will limit their movements to reduce the number of interactions with neighbours. Assuming these associations with neighbouring chicks are predominantly aggressive, we further expect their frequency to depend on chick competitiveness, that is, chick size.

## 2. Material and methods

### 2.1. Social environment and cross-fostering

The fenced-off colony in which this study took place is located in the outer harbour of Zeebrugge, Belgium (51°20′56.2″ N, 3°10′25.0″ E) and hosted 282 pairs of lesser black-backed gulls during the study year (2021). To experimentally create variation in social density within the colony, 221 U-shaped concrete blocks (preferred breeding sites for gulls in this colony) were unequally distributed across eight equally sized (850 m^2^) plots separated by 50 cm high plastic mesh fences in 2020 (for details see [32]). From the onset of egg laying onwards (around 25 April), we determined the laying date of each egg during three visits per week. Lesser black-backed gulls lay one egg every other day until the clutch of three eggs is complete. Experimental nests were selected based on peak laying dates, and after matching clutches by their respective laying dates, we cross-fostered complete clutches before any egg had hatched in the colony. The four high-density (HD) plots supported on average twice as many nests as the four low-density (LD) plots. In order to disentangle pre-natal from post-natal social density effects on chick movement activity, home range size and social associations, we cross-fostered a total of 76 nests between and within three LD plots and one HD plot (LD to HD: 16 nests; HD to LD: 28 nests; LD to LD: 22 nests; HD to HD: 10 nests). The single HD plot used in this study contained 43 nests. The average number of nests in the three LD plots was 20.68 ± 10.41. In order to account for within-plot variation and for the fact that only a part of the nests was part of the tracking, we also measured the social density by calculating the mean distance of each experimental nest to its three nearest neighbours before and after cross-fostering. As shown below, this measure (which was used in later analyses) coincided with the aforementioned manipulation of nest numbers at the plot level. All experimental chicks were individually marked at the first post-hatch check to allow accurate determination of their identity and age. At hatching, downy feathers were collected for molecular sex determination [45].

### 2.2. Ultra-wideband tracking and data treatment

Prior to UWB tagging, morphometric measurements of the head and tarsus length of each chick were taken to the nearest 0.1 mm using a digital calliper, and body weight was measured to the nearest 0.1 g using an electronic balance. To account for age differences between chicks, we calculated a body size index by using the residuals of a linear regression of tarsus length (relevant for movement) against age. We fitted a total of 81 chicks (from 51 nests) with UWB tags attached to an elastic wing harness. The chicks were between 15 and 25 days old and had a minimum body weight of 406 g, so that the total combined weight of the tracker and the harness (20.3 g) was between 3 and 5% of the chick’s body mass. Data were stored remotely by a network of receivers placed throughout the colony. These devices collected accurate spatial and temporal data based on the triangulation of very short pulses emitted by the tags relative to a grid of antennas placed throughout the colony. The tags were programmed to collect data every 30 s. The spatial accuracy of the UWB tags, estimated from 10 stationary tags placed throughout the colony, was 33 ± 14 cm.

Tracking lasted for three consecutive days, during which we did not visit the colony to avoid disturbance. A total of eight tags did not transmit data, and we omitted the tracking data of five chicks that were not associated with their foster nest location (i.e. because they were adopted or because their foster parents moved the territory). Of the remaining 68 tracked chicks, 59% were sibling pairs from 20 different nests. After tracking, we retrieved all deployed devices. Anomalous data points that occurred more than 0.5 m outside the fenced colony boundary (to account for spatial error of the tags) and with an apparent speed greater than 0.2 m s^–1^ (calculated as the distance between consecutive points divided by the time elapsed between those positions; electronic supplementary material, figure S1) were excluded from further analysis. Visual inspection of the data also revealed a small number of cases where certain *x*-coordinates were repeated, resulting in artificial straight lines. These data were also removed before analysis. Finally, as movement patterns and social associations could have been influenced by the presence of fences (colony boundary or separation of the different plots), we included a categorical variable in the analysis that took into account whether nests were located near a fence, defined as ‘at a distance of less than 1.32 m from a given nest’. This threshold value was calculated from the mean distance travelled by all chicks (0.91 ± 0.41 m). Only eight nests were found to be potentially affected by the presence of a fence.

### 2.3. Movement activity and home range size

To make the temporal resolution of the tracking data constant over time and comparable between chicks, we resampled the original data to a lower temporal resolution of 5 min for further analysis. To assess whether chick movements were consistent over the 3-day tracking period, we calculated the repeatability of the daily (square-root) mean movement of each chick over 1000 bootstrapped samples using the ‘rptR’ package version 0.9.22 [46]. As individual movement activity was highly consistent between days (repeatability with 95% CI: 0.71 (0.60–0.79)), we then calculated the distance travelled between successive spatial positions (resampled to 5 min, see above) averaged over the entire 3-day period (hereafter, referred to as ‘movement activity’) of all 68 chicks. To determine the home range size of each chick, we calculated the 95% kernel utilization distribution with reference bandwidth selection (hereafter, referred to as ‘home range size’).

### 2.4. Social association

To maximize the likelihood of detecting associations between chicks, a social network analysis was performed on the original (i.e. unsampled) UWB tracking data. A social association between two individuals was considered to take place when both occurred simultaneously at a distance of less than 60 cm (i.e. taking into account spatial resolution, see above) with time stamps rounded to the nearest 30 s (over 80% of the fixes were collected every 30 s, while in 10% of the cases, there were gaps of 30 s (60 s sampling interval) and in 10% of cases gaps of up to 150 s (180 s intervals); electronic supplementary material, figure S2). We then performed a social network analysis using the R package ggraph version 2.1.0 [47] to derive individual-level estimates of (i) degree centrality (i.e. the total number of individuals with whom a given chick had contact) and (ii) association strength (i.e. the total number of social associations of a given chick over the 3-day tracking period). Due to the limited availability of UWB tags, a maximum of two chicks per nest were tracked, and not all neighbouring chicks of a focal nest were tracked. Therefore, measures of degree centrality and association strength should be considered as proxies rather than absolute values, as interactions with untracked chicks could not be quantified.

### 2.5. Statistical analysis

To test whether movement activity and home range size were related to pre- and post-natal density, we fitted two linear mixed models with either the square-rooted movement activity or the square-rooted home range size as response variables. The following fixed effects were included: sex, age-corrected body size, mean distance to the three nearest neighbours (pre- and post-natal) as a proxy for breeding density, proximity to the fence (calculated as a categorical variable) and the interaction between pre- and post-natal distance. Plot ID was included as a random factor. The interaction was removed from the model if it was not significant. Similarity in mean movement behaviour between siblings was tested using Pearson’s product–moment correlation.

To test whether degree centrality was related to pre- and post-natal density, we fitted a generalized linear mixed model with degree centrality as the response variable and a Poisson error distribution. Sex, age-corrected body size, pre-natal average distance to the three nearest neighbours, post-natal mean distance to the three nearest tracked neighbours and proximity to the fence were included as fixed effects, and only plot ID was included as a random effect to reduce model overfitting (as 50% of nests had only one data point). To test whether association strength was related to pre- and post-natal density, we first log-transformed the data. We then fitted a linear mixed model with a Gaussian error distribution with log-transformed association strength as the response variable and a fixed and random model structure identical to the previous one.

Therefore, we estimated the coefficients, standard errors and test statistics using conventional statistics on the observed network data. However, data derived from social networks are inherently non-independent and therefore violate the assumption of data independence [48–51]. Therefore, to determine the impact and statistical significance of the observed effects of the different covariates on the response variables, they must be compared against a null model [48,49]. A node-based permutation approach, suitable for comparing network positions with node attributes [49,50], was used, in which node labels were randomized 10 000 times across the network, keeping edges and network structure constant. Node randomization was constrained within plots. Coefficients were recalculated after each permutation using the same models as above, which generated a null distribution for each covariate. Effects were considered statistically significant if the observed coefficient derived fell outside the 95% distribution.

The models were fitted using the ‘nlme’ package version 3.1-162 [52] and the ‘lme4’ package version 1.1-35.1 [53] in R (R Core Development Team 2022). Normality, independence and homoscedasticity were assessed by analysing the model residuals as well as via the ‘DHARMa’ package version 0.4.6 [54]. Package ‘ggplot2’ version 3.4.2 was used to visualize our results [55], and statistical significance was set at a critical α level of 0.05.

## 3. Results

In support of our density manipulation, the mean distance to the three nearest neighbouring nests of the subset of nests whose chick movement activity was subsequently measured with UWB tags, differed significantly between HD and LD plots before cross-fostering (HD = 2.96 ± 0.80 m; LD = 4.78 ± 2.26 m; χ^2^ = 12.37, *p* < 0.001) and after cross-fostering (HD = 2.96 ± 0.78 m; LD = 4.11 ± 1.66 m; χ^2^ = 4.68, *p* = 0.03). As the interaction between pre- and post-natal social distance was never significant, only the statistical results of the reduced models (i.e. after removal of the non-significant interaction) are reported in the text below. The results of the full models are presented in table 1.

**Table 1.** Full linear mixed models testing the effects of sex, age-corrected body size, pre- and post-natal mean distance to the three nearest neighbours (and their two-factor interaction in models on movement activity and home range size), and proximity to the fence on (i) movement activity, (ii) home range size, (iii) degree centrality and (iv) association strength. Nest ID nested within plot ID was included as a random factor. Test statistics refer to Chi-square values (1–2) and *z* scores (3–4) with 1 d.f. Significant effects (*p* < 0.05) are marked in bold.

	coefficient	SE	test statistic	*p*‐value
***1. movement activity***				
pre-natal distance to neighbours	0.02	0.03	0.47	0.49
post-natal distance to neighbours	0.18	0.04	26.52	**0.0001**
sex	−0.02	0.03	0.56	0.45
chick size	0.02	0.02	2.05	0.15
fence category	−0.10	0.06	2.54	0.11
pre-natal × post-natal	0.11	0.06	2.83	0.09
***2. home range size***				
pre-natal distance to neighbours	0.25	0.35	0.13	0.72
post-natal distance to neighbours	2.21	0.49	28.12	**0.0001**
sex	0.19	0.35	0.30	0.58
chick size	0.03	0.20	0.03	0.87
fence category	−1.67	0.77	4.74	**0.03**
pre-natal × post-natal	1.23	0.80	2.37	0.12
***3. degree centrality***				
pre-natal distance to neighbours	0.07	0.07	0.98	0.20
post-natal distance to tracked neighbours	−0.13	0.07	−1.79	0.07
sex	0.18	0.18	1.00	0.15
chick size	−0.05	0.08	−0.65	0.26
fence category	0.19	0.17	1.09	0.25
***4. strength interactions***				
pre-natal distance to neighbours	0.16	0.19	0.88	0.23
post-natal distance to tracked neighbours	−0.41	0.19	−2.18	**0.03**
sex	0.90	0.47	1.91	**0.03**
chick size	−0.30	0.21	−1.42	0.11
fence category	1.00	0.49	2.04	**0.05**

### 3.1. Movement activity and home ranges

**Figure 1.**
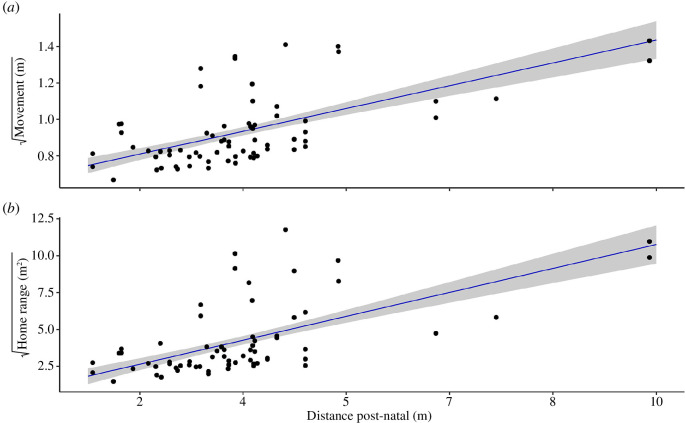
Relationship between mean distance to the three nearest nests (post-natal) and the square rooted (*a*) movement activity (m) and (*b*) home range size (m^2^) of ultra-wideband-tagged lesser black-backed gull chicks. The grey bar represents the standard error around the prediction. See text for details.

Chicks moved significantly less when the neighbouring nests were closer by (χ^2^ = 25.51, *p* < 0.0001, figure 1*a*), while no significant relationship was found with the pre-natal social distance (χ^2^ = 0.45, *p* = 0.50). Movement activity was strongly positively correlated between siblings from the same nest (*r* = 0.94, *p* < 0.001). Chicks reared in territories where neighbouring nests were closer also had smaller home ranges (χ^2^ = 27.31, *p* < 0.0001, figure 1*b*), while, again, no significant relationship was found with the pre-natal social distance (χ^2^ = 0.12, *p* = 0.72). Chicks also had smaller home ranges when their nest was located closer to a fence (χ^2^ = 4.18, *p* = 0.04). Similar to movement activity, home ranges were strongly positively correlated between siblings from the same nest (*r* = 0.92, *p* < 0.001).

### 3.2. Degree centrality and association strength

**Figure 2.**
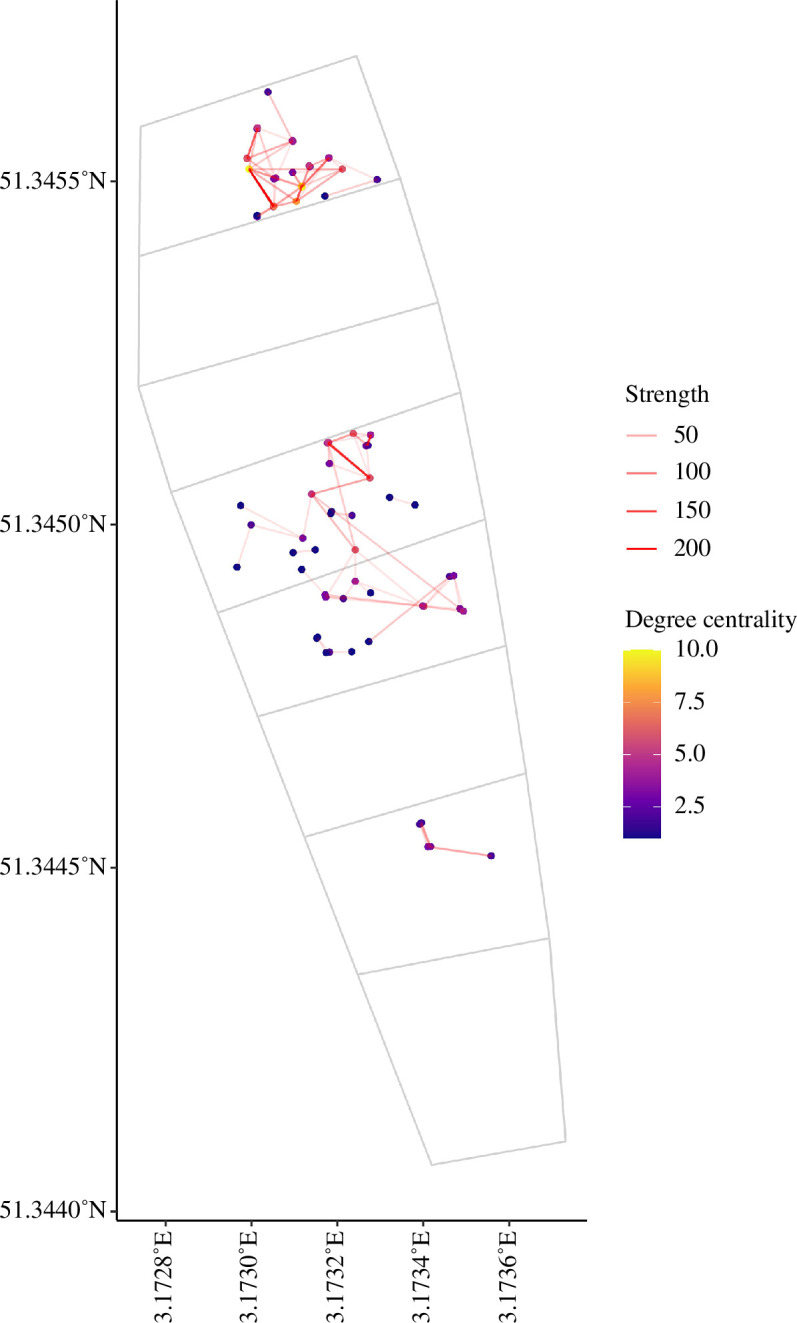
Spatial representation of the social network of 68 lesser black-backed gull chicks fitted with ultra-wideband tags. The dots represent the average spatial location of each chick during the 3 days of tracking, and their colour is related to the degree of centrality. The thickness of the lines connecting the chicks represents the strength of the social associations. The fences demarcating the colony and the different plots are shown as grey lines.

Chicks had an average of 2056 (range = 850–4013) social associations with their siblings, compared to only 60 (range = 1–571) associations with an average of 3 (range = 1–10) neighbouring chicks. Considering only associations with non-sibling chicks (figure 2), we found that none of the covariates had a significant effect on degree centrality (table 1). Although none of the covariates had an effect on the number of unique contacts, the total number of contacts (association strength) decreased as the distance of their nest to the three nearest tracked neighbours decreased (table 1). Males had more contacts than females (males: 3.12 ± 0.65; females: 2.62 ± 0.62, *p* = 0.03), and chicks from nests close to a fence also had more social associations than those from nests farther from the fence (close to fence: 3.67 ± 0.73; far from fence: 2.67 ± 0.60, *p* = 0.05). No significant relationships were found between pre-natal social distance and degree centrality or association strength (table 1).

## 4. Discussion

Many seabirds, such as gulls of the genus *Larus*, breed in large numbers in close proximity to each other. Such colonial breeding is associated with high levels of species-specific aggression between adults, for example when establishing territories. In lesser black-backed gulls, chicks also experience high (sometimes lethal) levels of aggression from both neighbouring adults and chicks [27–29,31]. These chicks would therefore be expected to adapt their behaviour to help them cope with hostile social conditions early in life, for example, by adjusting their movement activity to reduce the number of encounters with conspecifics. We found that individuals reared in close proximity to neighbours moved less and occupied smaller home ranges than individuals reared further away from other nests. Despite this reduced movement activity, the total number of individuals with whom a given chick had contact, and the total number of social associations, were still higher in high-density areas. Below, we discuss how our results can be interpreted as adaptations to the social environment during early life and speculate why none of the measured traits were associated with breeding density during the pre-natal phase.

### 4.1. Post-natal density and movement activity

As we followed individuals between 15 and 25 days of age, whereas lesser black-backed gull chicks are able to move from 5 to 7 days of age, the reduced movement activity and smaller home ranges in closer proximity to neighbours may be due to aversive feedback learning [56,57]. Indeed, the chicks were likely to have previously experienced aggressive interactions when crossing a neighbour’s territory. Such reduced movement behaviour is consistent with our previous finding that chicks reared in high-density areas also show reduced exploration activity, as measured in an experimental open-field test [32]. Both of these findings suggest conflict avoidance strategies in densely populated areas, where encounters with neighbours are more likely. While such behaviour may be considered adaptive in the short term, it may inhibit the later expression of exploratory behaviour. This, in turn, may have negative consequences for memory, learning or spatial cognition [58,59]. As individuals are thought to integrate environmental information acquired early in life into a channelled phenotype in adulthood [60,61], post-natal social densities may also have longer-term behavioural consequences. For example, reduced spatial cognition due to limited exploratory behaviour early in life, if sustained, could have negative effects on the ability to track the location of food sources when foraging. However, this remains highly speculative, as little is currently known about the extent to which movement and exploration in early life influence spatial cognition later in life—and to what extent this is species-specific. For example, in cliff breeders, offspring have little ability to move, while some of these species still travel long distances as adults during their annual cycle, requiring strong navigational and spatial skills.

### 4.2. Post-natal density and social associations

Despite their reduced movement and smaller home ranges, chicks reared at higher densities showed a higher number of associations with neighbouring chicks. However, we did not find a significant effect of post-natal density on degree centrality. Taken together, this suggests that individuals from nests that were closer to others generally had more contacts than chicks from more distant nests, but not necessarily with more unique individuals. Thus, chicks reared at high densities may not be able to reduce social interactions to the level of those reared at low densities. Nevertheless, the number of interactions would most likely have been much higher if the chicks had moved around as much as the chicks from lower-density areas. Even in the high-density areas, most social associations were between siblings, with less than 4% involving chicks from neighbouring territories. This suggests that most of the early-life social environment is shaped by sib–sib interactions. While this is an interesting finding, it should be interpreted with caution as we were only able to track about 50% of the nests, and hence their chicks. Therefore, not all chick–chick interactions (and any chick–adult interaction) were registered.

The level of social association differed between male and female chicks, favouring males. This suggests that it may be determined by the competitiveness of a given chick. In lesser black-backed gulls, males tend to be larger than females, and this is also the case in our study population (R. Salas 2024, unpublished data). As male chicks are able to win conflicts with neighbouring chicks due to their larger size, they may be less likely to avoid them. Another possibility is that sex differences in social associations are determined by testosterone levels [62] rather than size. Testosterone mediates territorial aggression in adult gulls [36], and circulating testosterone has been shown to also increase territorial aggression in chicks of the black-headed gull *Chroicocephalus ridibundus* [62–64]. However, these conclusions, too, remain highly speculative as our data only indicate the spatial proximity of neighbouring chicks and further research is needed to determine the nature of these contacts.

### 4.3. No evidence of pre-natal effects

The relationships discussed above between movement, ranging, social associations and density experienced by chicks early in life confirm that parents, through their choice of breeding site, strongly determine the social context and behavioural phenotype of their offspring. However, chicks do not necessarily hatch unprepared for this environment, as mothers can allocate hormones or other components to their eggs that can prepare their offspring for the conditions they are likely to encounter after hatching [40–42]. In support of this, pre-natal breeding density significantly predicted chick exploration behaviour in the open field test with chicks from the same colony, including individuals from this study [32], with pre- and post-natal social environment contributing almost equally to chick exploration behaviour. While this suggests that offspring may indeed benefit from pre-natal programming when post-natal conditions match pre-natal conditions, we can only speculate why we did not find a similar pre-natal effect when we directly tracked chicks in their post-natal social environment. One reason may be that the current, post-natal environment determines how the chicks actually *can* move, whereas an open field test without potentially aggressive attacks reveals the potential of how the chicks *might* move. In other words, maternal programming of chick behaviour, rather than the expression of the trait itself, may influence the disposition and potential of a chick. While this is still speculative at this stage, it would be consistent with pre-natal organizational effects of maternal testosterone exposure [65], and indeed there is evidence that maternal testosterone deposition is shaped by social density during egg laying [38,66–69].

## 5. Conclusions

Using an innovative tracking design in a ground-based colony, we have shown that under socially dense conditions, the space available for chicks to roam freely is limited to the immediate vicinity of the nest site. Thus, by choosing a particular nest site, parents have a strong influence on the early social environment of their chicks, and possibly also on their behavioural development. In order to better understand parental nest site selection, or even colonial breeding in general, these offspring related aspects need to be taken into account. Interestingly, social network analysis showed that contacts with neighbouring chicks were very rare, again indicating the agonistic nature of inter-nest interactions. Further research is now needed to determine whether restricted movement activity is adaptive, for example, by reducing levels of aggression, or whether restrictions on mobility and exploration early in life may have lasting negative effects on cognitive development.

## Data Availability

All data and code are accessible through [70]. Electronic supplementary material is available online [71].
